# Innate immunity and hepatitis C virus infection: a microarray’s view

**DOI:** 10.1186/1750-9378-7-7

**Published:** 2012-03-26

**Authors:** Luigi Buonaguro, Annacarmen Petrizzo, Maria Lina Tornesello, Franco M Buonaguro

**Affiliations:** 1 Molecular Biology and Viral Oncogenesis Unit, Istituto Nazionale Tumori “Fond. G. Pascale”, 80131, Naples, Italy

**Keywords:** HCV, Innate immunity, Pattern recognition receptors (PRRs), Interferon stimulated genes (ISGs), Systems biology

## Abstract

Hepatitis C virus (HCV) induces a chronic infection in more than two-thirds of HCV infected subjects. The inefficient innate and adaptive immune responses have been shown to play a major pathogenetic role in the development and persistence of HCV chronic infection. Several aspects of the interactions between the virus and the host immune system have been clarified and, in particular, mechanisms have been identified which underlie the ability of HCV to seize and subvert innate as well as adaptive immune responses. The present review summarizes recent findings on the interaction between HCV infection and innate immune response whose final effect is the downstream inefficient development of antigen-specific adaptive immunity, thereby contributing to virus persistence.

## Hepatitis C virus: a brief overview

Hepatitis C virus (HCV) is a Hepacivirus of the Flaviviridae family, mainly involved in hepatic disorders, including chronic hepatitis which may progress to cirrhosis in about 10–20% of cases and further to hepatocellular carcinoma (HCC) in 1–5% of cirrhotic patients [1]. Furthermore, HCV has also been implicated as one of the major etiologic factors for type II Mixed Cryoglobulinemia (MC), an autoimmune disease that may evolve into an overt B-cell non-Hodgkin’s lymphoma (NHL) in about 10% of MC patients [2-4].

HCV is an enveloped positive-strand RNA virus. Six major HCV genotypes and more than 100 subtypes have been so far identified [5-7]. HCV genomic RNA contains a single open reading frame flanked by 5^′^ and 3^′^ untranslated regions (UTRs) [8,9], which encodes for a single large polyprotein, processed by cellular and viral proteases to produce structural as well as non structural proteins [9,10].

The core (C) and envelope proteins (E1 and E2) are structural components of the infectious particle [11], whereas the non structural (NS) proteins are required for RNA replication (NS3–NS5B), particle assembly (p7 and NS2) and protein maturation (NS2 and NS3/4A complex) [10,12].

HCV entry into hepatocytes is mediated by one of the following putative receptors, such as the CD81 tetraspanin [13], the scavenger receptor class B type I [14], the tight junction proteins claudin [15,16] and occludin [17], the latters conferring species specificity.

## Innate immune response to HCV

As for all microbial infections, innate immune response plays a critical role in the control and resolution of HCV infection providing signals for the efficient priming of the adaptive branch of immune response [18,19].

In particular, the innate immunity is important in HCV infection to control viral dissemination and replication in order to allow an adequate downstream development of antigen-specific humoral as well as cellular responses [20].

During the early phase of HCV infection, the viral RNA load increases in the first few days and remains high throughout the incubation period, which lasts for up to 10–12 weeks post-infection [21,22]. In such early stage, large amounts of type I interferons (IFN-α, IFN-β) may be produced by HCV-infected hepatocytes as well as dendritic cells (DCs) to control viral replication [23].

Besides producing type I IFN, DCs represent the key cell compartment of innate immunity, orchestrating the quality and potency of downstream adaptive immune response. They are professional antigen presenting cells (APCs) able to uptake and process viral antigens, as well as release cytokines to efficiently prime both CD4+ helper T cells and CD8+ cytotoxic T lymphocytes (CTLs) [24].

In particular, the subset of plasmacytoid DCs (pDCs) is considered the front line in antiviral immunity owing to their capacity to rapidly produce high amounts of type I interferon in response to viruses, upon recognition of viral components and nucleic acids through Toll-like receptor (TLR) 7 and TLR9 [25,26]. To further support such role, several reports show a decreased frequency of pDCs in peripheral blood of patients with chronic HCV infection and impaired production of IFN-α by pDCs from HCV patients [27-29]. Also, myeloid or conventional DCs (cDCs) are programmed to produce IFN-α in response to viral infection upon interaction between viral double-stranded RNA-like molecule polyinosinic:polycytidylic acid (poly I:C) and TLR3 [30]. Moreover, cDCs produce high amounts of cytokines, such as IL-12, which has been shown to play an important role in stimulating IFN-γ production from activated T cells, inducing the development of type 1 (Th1) protective immune response [31,32]. Indeed, a recent study showed that an increased number of cDCs during acute HCV infection may be associated with viral clearance, whereas a loss in the number of cDCs may increase the risk for development of chronic HCV infection [33,34].

Also natural killer (NK) cells are potent antiviral effectors due to their contribution to virus elimination via direct killing of infected cells and cytokine production [35]. Genetic factors appear to contribute to the level of NK cell responsiveness, as shown by the presence of individual killer cell Ig-like receptor/human leukocyte antigen (KIR/HLA) compound genotypes correlated with HCV clearance [36]. In particular, given that the interaction between KIRs expressed on NK cells and HLA expressed on target cells plays a key role in NK cell activation, it has been suggested that such genotypes are characterized by a higher sensitivity of NK cells with a faster degranulation and IFN-γ release in vitro [37].

Nevertheless, innate immune response to HCV may also be detrimental inducing immunopathological effects on the liver. NK-mediated killing of HCV-infected hepatocytes and secretion of proinflammatory cytokines may cause liver damage, stimulating cDCs to produce high amount of IFN- γ which, subsequently, activates hepatic macrophages to enhance local inflammation [38]. All such cascade of events contributes to the pathogenesis of liver disease [39].

The overall data demonstrate the complex, contradictory and evolving equilibrium between HCV and host innate immunity, whose result will lead to completely different clinical outcomes ranging from resolution to chronic viral infection.

## Pattern recognition receptors in sensing virus infection

The innate immune response to virus infection is activated when conserved motifs of microbial origin, known as pathogen-associated molecular patterns (PAMPs) are recognized by cell pattern recognition receptors (PRRs) [40].

The 3 major classes of PRRs include Toll-like receptors (TLRs), RIG-I-like receptors (RLRs), and nucleotide oligomerization domain (NOD)-like receptors (NLRs) [41-43]. Viral engagement of TLRs and RLRs leads to the activation of transcription factors, such as the IFN regulatory factors (IRFs) and NF-κB, which in turn may lead to the activation of IRF3 target genes, type I IFN, and proinflammatory cytokines [44].

So far, the role of NLRs in sensing RNA viruses is still unclear and they are primarily thought to be activated by intracellular stress signals (i.e. damage-associated molecular patterns, DAMPs) [45]. In this regards, DAMPS derived from HCV infected hepatocytes may play a critical role in promoting liver inflammation with immunopathological effects.

Innate immune cells (i.e. monocytes, neutrophils, dendritic cells) are rapidly activated upon recognition of infecting agents by a wide range of PRRs. Among them, the Toll-like receptors are members of the interleukin-1 receptor (IL-1R) superfamily [46,47], characterized by a leucine-rich repeat (LRR) domain in the extracellular region and an extracellular Toll/IL-1R (TIR) domain [48].

So far, 11 TLRs have been identified. TLR1, TLR2, TLR6 and TLR10 are closely related to the TLR2 subfamily, whereas, TLR7, TLR8 and TLR9 are closely related to the TLR9 subfamily [49]. Each TLR has specific ligands, which allow the host to sense a wide diversity of pathogens [50]. As a result of TLR stimulation, proinflammatory cytokines are released that activate the host immune response [51-53].

Most TLRs (except for TLR3) share a common signalling pathway, via the adaptor molecule, myeloid differentiation primary response protein 88 (MyD88) [54-56]. TLRs recruit MyD88 via TIR domains, then MyD88 binds the IL-1R-associated kinase (IRAK) and the signal is propagated to tumor necrosis factor (TNF) receptor-associated factor-6 (TRAF6), for activation of NF-kB and mitogen-activated protein kinases (MAPK). A second pathway involves the TIR-associated protein (TIRAP)/MyD88-adaptor-like (Mal) for TLR1/2, TLR2/6 and TLR4 signalling [57,58].

On the contrary, TLR3 signalling is independent of MyD88 and is mediated by TIR-domain containing adaptor inducing IFN-b (TRIF)_toll-like receptor adaptor molecule I (TICAM I) with induction of interferon-regulatory factor-3 (IRF-3) transcription factor and subsequent production of IFN-β [59].

The RIG-I-like receptors (RLRs) are sensors of viral RNA, consisting of three members: RIG-I, melanoma differentiation antigen 5 (MDA5) [60] and laboratory of genetics and physiology-2 (LGP2) [61-64]. RLRs are expressed in the cytoplasm of most cells, including hepatocytes, representing good candidates as primary intracellular sensors of HCV infection. Both RIG-I and MDA5 contain 2 N-terminal caspase activation and recruitment domains (CARD) [65]. Moreover, all the RLRs have a DExD/H RNA helicase domain and bind to RNA ligands [66]. In addition, RIG-I has a repressor domain that interacts with the CARD domains to maintain the receptor in a non-active conformation in the absence of infection [67]. LGP2 lacks the CARD domains and may function as a regulator of RLR signalling [68]. RIG-I senses non-self double-stranded RNAs (dsRNAs) with free 5^′^-triphosphates and is recruited to the mitochondrial surface, where interacts with MAVS (mitochondrial antiviral signalling protein; also known as IFN-β promoter simulator 1 (IPS-1), virus-induced signaling adapter (VISA), and CARD adaptor inducing IFN-β (Cardif)) on the outer mitochondrial membrane. MAVS is a CARD protein and an essential adaptor for RLR signalling [69]. The interaction between RIG-I and MAVS results in the activation of the transcription factors, interferon regulatory factor-3 (IRF-3) and nuclear factor-kB (NF-kB) with subsequent transcription of IFN-β [70].

Overall, the data show that, regardless the class of PRRs engaged upon viral recognition, the activated pathways in cells of the innate immunity converge to induce the production of type I IFN with highly effective anti-viral activity.

## IFN signalling during HCV infection

**Figure 1:**
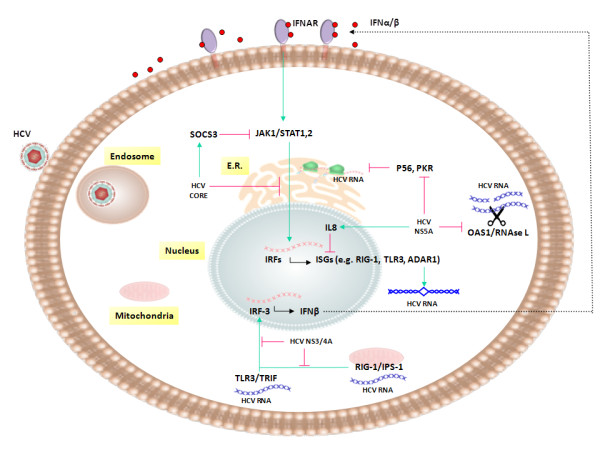
**Modulation of innate immune response by HCV.** Effects of different HCV factors, RNA or proteins, on cellular mediators of innate immune anti-viral activity are schematically represented.

The production of IFN-β resulting from HCV infection leads to activation of the JAK (Janus kinase)/STAT (signal transducer and activator of transcription) signalling pathway with the expression of interferon-stimulated genes (ISGs) (Figure 1) [71,72].

Among others, an increased expression has been described for the 2^′^-5^′^-oligoadenylate synthase 1 (OAS1)/RNAse L system, which degrades viral and cellular RNA [73], and the RNA-specific adenosine deaminase acting on RNA 1 (ADAR1), which converts adenosine residues into inosine residues in dsRNA [74], thereby mutating and destabilizing secondary viral RNA structures [75]. Similarly, induction of other ISGs, such as P56 [76] and protein kinase R (PKR) [77], which inhibit translation of viral and host RNAs [78], has been reported. Such induction of ISGs will ultimately amplify the IFN response, in a loop fashion, given that some pattern recognition and signalling molecules are ISGs per se, such as RIG-I, TLR3 and TRIF, whose final outcome is the production of IFN-β (Figure 1) [79].

In this complex framework, HCV establishes a chronic persisting infection when is able to disrupt the host immune response and to evade antiviral defenses. A major strategy employed by HCV to subvert the host innate immune response is to undermine IFN antiviral activity [80] as well as functions of innate immune cells.

Regarding the IFN activity, the main targets of HCV are represented by the PAMP signalling pathways leading to IRF-3 activation, the IFN-α/β receptor signalling pathway and the ISG effector proteins (Figure 1). In particular, HCV NS3/4A protein cleaves the adaptor molecules TRIF and IPS-1, thereby blocking TLR3 and RIG-I signalling pathways [81]. Moreover, the HCV core protein interferes with JAK/STAT signalling and ISGs expression by several strategies, including 1) inhibition and degradation of STAT1 [82]; 2) induction of the suppressor of cytokine signaling 3 (SOCS3) and protein phosphatase 2A (PP2A), which are an inhibitor of the JAK/STAT pathway [83] and a reducer of the transcriptional activity of ISG factor 3 (ISGF3) [84], respectively; 3) inhibition of ISGF3 binding to IFN-stimulated response elements (ISRE).

Furthermore, several HCV proteins directly interfere with the function of ISGs. Indeed, functional genomics analyses have shown that the NS5A protein induces a general attenuation of ISG expression via an increased secretion of IL-8 [85] and a subsequent modulation of IFN functions. In support of such inhibitory mechanism, serum levels of IL-8 have been found elevated in patients with chronic hepatitis C. In addition, NS5A inhibits 2^′^-5^′^ oligoadenylate synthetase (2′-5′ OAS) and PKR function [86] and E2 acts as decoy target to PKR [87].

Even though such escape strategies still need to be validated in vivo, the available data strongly suggest that HCV has established redundant means to cope with the host IFN response.

## Interactions between HCV and cellular compartments of innate immunity

More recently, several reports have suggested that HCV itself may actively suppress the host immune response by inhibiting the function of innate immune cells.

### NK cells

NK cell activation, during the early phase of HCV infection, is involved in viral eradication, whereas direct suppression of NK cells may be implicated in HCV chronic persistence [88]. It has been reported that the binding of HCV E2 protein to the CD81 receptor on NK cells inhibits their function and IFN-γ production [89,90]. Such results, however, are still controversial and their biological significance in vivo needs to be verified, considering that HCV E2 does not efficiently crosslink CD81 on NK cells when it is part of infectious virions, and NK cell function is not impaired after in vitro exposure to high concentrations of cell culture–produced HCV [91].

The HCV core protein induces an up-regulation in the expression of major histocompatibility complex (MHC) class I molecules on the surface of hepatocytes by increasing the expression of transporter associated with antigen processing 1 (TAP1). The resulting suppression of NK cells activation and cytotoxic activity significantly contribute to HCV persistence [92].

In addition, it has been reported that NK cells from chronic HCV-infected individuals show an increased expression of CD94/NKG2A inhibitory receptor, as well as production of immunosuppressive IL-10 and TGF*β*. The sum of these effects is the functional impairment of NK cells to activate DCs and, ultimately, to generate Th1 CD4+ T cells [93].

### DC cells

DC response to HCV in the early stage of infection is crucial in determining the outcome of the disease and several studies indicate that chronic HCV-infected individuals show an impaired function of DC subsets.

The frequency of circulating pDCs [94], as well as their ability to produce IFN-α upon in vitro stimulation [95] are reduced in chronic HCV patients and different mechanisms have been proposed. HCV core and NS3 proteins have been shown to activate in vitro monocytes via TLR2 to produce TNF-α, which in turn inhibits IFN-α production and induces pDCs apoptosis [96]. Alternatively, HCV may directly inhibit IFN-α production by pDCs in vitro [97].

Similarly, maturation and functional differentiation of cDCs are altered in HCV infection, with decreased IL-12 and increased IL-10 production in vitro [98,99], possibly resulting in insufficient T cell priming and delayed HCV-specific T cell responses. However, the impaired allostimulatory capacity of cDCs in chronic HCV patients is still contradictory, being described in some [100-102] but not all studies [103].

For both subsets of DCs, indeed, functional defects have been observed in vitro upon stimulation with individual HCV proteins but do not reflect an immune compromised status in chronic HCV patients, who show normal responsiveness to other viruses or recall antigens (reviewed in [104,105]). Therefore, the impaired efficacy of DCs compartment (i.e. pDCs) in chronic HCV patients would not be due to a “primary” dysfunction of such cells in producing type I IFNs but, more likely, to a “secondary” non-responsiveness of target HCV-infected hepatocytes, given the capacity of HCV to inhibit the IFN-stimulated signal pathway.

**Figure 2:**
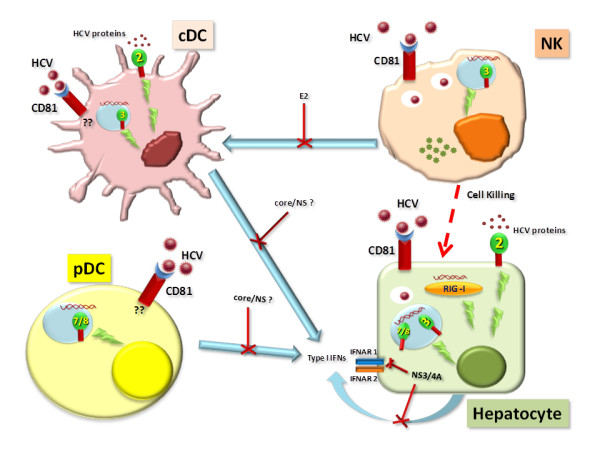
**Interactions between HCV and target cells.** Effects of the HCV infection on hepatocytes as well as cells of the innate immunity have been described. Inhibitory strategies (confirmed and contradictory) employed by individual HCV proteins have been indicated.

Overall data suggest that HCV interacts with and affects the function of different actors of the innate immunity. HCV interference is diverse with regard to cellular levels, targets and outcomes, however, the overall disruption of the coordinated activity of the innate immune response results in deficient adaptive immune response and prevention of pathogen elimination (Figure 2).

## HCV disease: a microarray’s view

The interplay between HCV and innate immunity can nowadays be addressed and studied by systems biology approaches which provide detailed level of investigation to better and fully analyze the network of interactions within virus and innate immunity. Conversely to traditional “reductionist” approach, indeed, the paradigm of systems biology is to look at a biological system as a whole, evaluating interactions among biological elements and their relationship with the surrounding environment. Systems biology has been increasingly applied to oncology [106-108], autoimmunity and infections [109,110] and only recently to vaccinology [111-113].

Microarray analyses of gene transcriptional profiles have been performed to identify molecular signatures of the innate immunity compartment related to HCV infection (Table 1).

**Table 1:** Differentially expressed genes identified in listed studies on liver tissues from chronic HCV patients

** GENE NAME**	**DESCRIPTION**
**Folkers et al.[**[114]**]**	
IFNAR1	Interferon-alpha/beta receptor alpha chain Precursor
IRF1	Interferon regulatory factor 1
LAIR1	Leukocyte-associated immunoglobulin-like receptor 1 Precursor
LEAP2	Liver-expressed antimicrobial peptide 2 Precursor
LILRA1	Leukocyte immunoglobulin-like receptor subfamily A member 1 Precursor
LILRA4	Leukocyte immunoglobulin-like receptor subfamily A member 4 Precursor
LILRA5	Leukocyte immunoglobulin-like receptor subfamily A member 5 Precursor
LILRB2	Leukocyte immunoglobulin-like receptor subfamily B member 2 Precursor
LILRB3	Leukocyte immunoglobulin-like receptor subfamily B member 3 Precursor
LILRB4	Leukocyte immunoglobulin-like receptor subfamily B member 4 Precursor
MAP4K2	Mitogen-activated protein kinase 2
PSMB4	Proteasome subunit beta type-4 Precursor
RFX5	DNA-binding protein RFX5
TRIM22	Tripartite motif-containing protein 22
TRIM34	Tripartite motif-containing protein 34
**Khalid et al.[**[138]**]**	
CCR4	Chemokine receptor 4
CXCL10	Chemokine ligand 10
HLA DQβ1	Major histocompatibility complex II DQ beta1
HLA DQχ1	Major histocompatibility complex II DQ alpha1
IFI16	IFNα induced protein 16
IGLL	Immunoglobulin lambda locus
IL2RA	Interleukin 2 receptor alpha
LGALS3	Lectine galactoside binding soluble 3
SCYA19	Small inducible cytokine subfamily A
SGK	Serum/glucocorticoid regulated kinase
**Blackham et al.[**[139]**]**	
CXCL1	Chemokine ligand 1
CXCL16	Chemokine ligand 16
CXCL2	Chemokine ligand 2
CXCL3	Chemokine ligand 3
CXCL5	Chemokine ligand 5
CXCL6	Chemokine ligand 6
IL-8	Interleukin 8
IRF1	Interferon regulatory factor 1
IRF9	Interferon regulatory factor 9
MASP1	Mannan-binding lectin serine protease 1
MBL2	Mannose-binding lectin (protein C) 2, soluble
MX1	Interferon-induced GTP-binding protein Mx1
SOCS2	Suppressor of cytokine signaling 2
SOCS3	Suppressor of cytokine signaling 3
**De Giorgi et al. [**[128]**]**	
B2M	Beta 2 microglobulin
CD7	CD7 molecule
HLAF	Major histocompatibility complex, class I, F
IFI16	IFNα induced protein 16
IFI27	IFNα induced protein 27
OASL	2′-5′-oligoadenylate synthetase-like protein
PSMB9	Proteasome subunit beta type-9
PSME2	Proteasome activator subunit 2
STAT	Signal transducer and activator of transcription 1, 91 kDa
TAP1	Transporter 1, ATP-binding cassette, sub-family B (MDR/TAP)

It has been recently shown that specific immune genes are significantly increased in HCV cirrhotic liver as compared to control normal tissue [114]. Such genes include IRF1, tripartite motif-containing 22 (TRIM22), and multiple leukocyte immunoglobulin-like receptors (LILRA1, LILRA4, LILRA5, LILRB2, LILRB3 and LILRB4), which have been reported to play a role in the virus – host interaction.

IRF1 is a critical transcriptional regulatory factor that modulates ISG expression and has been shown to regulate HCV subgenomic replicon activity in cultured hepatoma cells [115]. Interestingly, polymorphisms in the IRF1 promoter have been associated with a better response to IFN-α therapy in patients with chronic HCV infection [116].

TRIM22 belongs to the tripartite motif family of proteins which have been associated with innate immune response to viruses, inhibiting viral replication [117].

Moreover, multiple leukocyte immunoglobulin like receptors (LILRs) are known to be expressed on myelo-monocytic cells and can influence both the innate and acquired immune response. In particular, LILRB2 has been previously reported to be up-regulated also in HIV patients and may impair the antigen presentation function of monocytes [118], whereas, LILRB4 has been shown to impair antigen presentation and T cell recruitment modulating the expression of proinflammatory cytokines [119].

A different study showed a total of 524 genes differentially expressed in “advanced HCV” as compared to non viral hepatitis, with 466 up-regulated genes and 58 down-regulated genes [120]. The most affected biological functions observed in “advanced HCV” include the canonical pathways of calcium signalling, hepatic fibrosis/stellate cell activation and actin cytoskeleton signalling. Moreover, many differentially expressed genes involved in the pathways of immune system, fibrosis, proliferation, cell growth, and apoptosis have been found to be up-regulated according to previously published data [121-123]. The majority of such genes are involved in the pathway of the immune and inflammatory response, including: class II major histocompatibility complex HLADQa1, HLADRa1, chemokines and chemokine receptors [124].

Moreover, a microarray analysis performed on Huh7 hepatocarcinoma cell line demonstrated that infection with HCV JFH-1 viral particles alters the expression of host genes involved in cellular defense mechanisms that protect the cell against infection and oxidative stress which, in turn, determine the fate of cellular survival [125]. Furthermore, HCV JFH-1 infection is able to stimulate the expression of proinflammatory antiviral response genes, including those involved in type I and II interferon responses (e.g. IRF1, IRF9, and myxovirus (influenza virus) resistance 1, interferon-inducible protein p78 (mouse) (MX1) genes), the complement cascade (e.g. mannose-binding lectin (MBL) 2 and mannose-binding protein associated serine protease (MASP) 1 genes), and the production of proinflammatory chemokines and cytokines (e.g. IL-8 and CXC chemokine ligand (CXCL) 1,−2,−3,−5,−6, and−16 genes) [126]. Increased expression of genes encoding for negative regulators of the interferon response has been also observed, including several members of the SOCS gene family (e.g., SOCS2 and−3 genes) [127].

In this framework, our group evaluated differential gene expression by microarray analysis on liver biopsies obtained from chronic HCV and control negative patients [128]. Unique gene signatures were identified and, in particular, the HCV infected liver tissue showed strong up-regulation of genes involved in antigen presentation, protein ubiquitination, interferon signaling, IL-4 signalling, bacteria and viruses cell cycle and chemokine IL-4 signalling pathways.

Data analysis focused on the expression levels of specific genes related to the innate immunity pathway showed a strong up-regulation of genes involved, at multiple levels, in the pathway of Type I IFN signalling, including the STAT1 transcription factor and the downstream regulated genes (ISGs), in agreement to studies from other groups [129].

Among the observed up-regulated ISGs, gamma-interferon-inducible protein 16 (IFI16), interferon alpha-inducible protein 27 (IFI27) and 2^′^-5^′^-oligoadenylate synthase-like (OASL) were identified. In particular, IFI16 controls cellular proliferation by modulating the functions of cell cycle regulatory factors including p53/TP53 and the retinoblastoma protein (pRb) [130,131]. IFI27 is known to promote cell death, triggering an IFN-induced apoptosis with robust release of cytochrome C from the mitochondria and activation of BCL2-associated X protein (BAX) and caspases 2, 3, 6, 8 and 9 [132]. Furthermore, the 2^′^-5^′^-oligoadenylate synthase-like protein (OASL) has been shown to bind double-stranded RNA and DNA, suggesting a role in anti-viral activity [133].

Moreover, MHC components of antigen processing and presentation such as HLA-F, Beta-2-microglobulin (B2M), CD7 and TAP1, have been found up-regulated in HCV-positive liver tissue. In particular, TAP1 is involved in the transport of antigens from the cytoplasm to the endoplasmic reticulum for association with MHC class I molecules [134]. Indeed, it is well known that HCV core protein enhances MHC class I molecule function, by increasing the expression of TAP1, thus contributing to HCV persistence by suppressing the cytotoxic activity of NK cells [135].

Furthermore, data analysis focused on the expression levels of specific genes related to the innate immunity pathway show a relevant activation trend of the flagellin-dependent TLR5, associated with the activation of IRAK1 (variant 3) and decreased level of IL-10 and IL-1b (submitted for publication).

Such overall data shed light on specific pathogenetic mechanisms and gene signatures involved in HCV-related disease and suggest the relevant role of innate immunity in progression of HCV infection. Furthermore, the analysis of relevant pathways and specific genes involved in HCV progression to cancer may have a relevant impact on the early identification of “progressors” to select for appropriate therapeutic actions.

Indeed, despite advances have been made in the treatment of HCV chronic infection with the combination of pegylated interferon (PEG-IFN) and ribavirin, treatment failure still occurs in about half of the patients and prediction of treatment response would be of great value.

In this perspective, several studies employed gene expression profiling analysis to investigate the molecular basis for treatment failure in HCV chronic infection.

In particular, a systems biology approach using high-throughput technologies, such as complementary DNA microarrays combined with mathematical modeling, was applied to identify a liver gene signature to predict sustained virological response to PEG-IFN plus ribavirin in patients with HCV chronic hepatitis [136,137].

To this aim, expression profiling analysis was performed on liver biopsy specimens taken before therapy and gene expression levels were compared among 15 nonresponders (NR), 16 responders (R), and 20 healthy subjects.

Eighteen genes were differentially expressed between responders and nonresponders. Up-regulation of a specific set of IFN-responsive genes predicted poor response to therapy, suggesting a possible rationale for treatment resistance.

In particular, upregulation of the following 8 genes showed the most consistent ability to classify NR and R subjects: ISG15, activating transcription factor 5 (ATF5), IFN-induced protein with tetratricopeptide repeats (IFIT1), MX1, ubiquitin-specific protease 18 (USP18), dual specificity phosphatase 1 (DUSP1), cyclin E binding protein (CEB1) and 40S ribosomal protein S28 (RPS28). Overall, the study showed that different innate IFN response to HCV infection may significantly impact on the responsiveness to PEG-IFN plus ribavirin therapy and identify NR and R patients [137].

## Conclusions

The host immune response plays a critical role in HCV infection because of its potential to contribute not only to viral clearance but also to liver injury. HCV attenuates both innate and adaptive immune responses, thereby reducing the viral clearance as well as the degree of immune-mediated liver injury, allowing coexistence of both virus and host. Key questions for future studies remain for nearly every aspect of the host immune response; so far, the pathogenetic mechanisms involved in progression to distinct HCV-related malignant tumors are still ill defined.

However, the analysis of the innate immune pathways involved in HCV chronic infection would help elucidating the possible mechanisms leading to HCV related cancers, such as HCC or B-cell NHL.

Future studies focused on the analysis of relevant pathways and specific genes involved in HCV infection and progression to cancer would have a relevant impact on the understanding of HCV-related carcinogenesis (HCC and/or B cell NHL) as well as on the management of HCV-infected subjects, making easier the identification of “progressors” to select for appropriate preventive/therapeutic actions.

## Competing interests

The authors declare that they have no competing interests.

## Authors’ contributions

LB and AP drafted the manuscript. MLT participated in the draft of the manuscript. FMB conceived of the study and participated in its design and coordination and helped to draft the manuscript. All authors read and approved the final manuscript.
